# cRegions—a tool for detecting conserved cis-elements in multiple sequence alignment of diverged coding sequences

**DOI:** 10.7717/peerj.6176

**Published:** 2019-01-10

**Authors:** Mikk Puustusmaa, Aare Abroi

**Affiliations:** 1 Department of Bioinformatics, Institute of Molecular and Cell Biology, University of Tartu, Tartu, Estonia; 2 Institute of Technology, University of Tartu, Tartu, Estonia

**Keywords:** Embedded functional element, Cis-element, Codon usage bias, Alphavirus, Cis-acting sequence, Viruses, Multiple sequence alignment analysis

## Abstract

Identifying cis-acting elements and understanding regulatory mechanisms of a gene is crucial to fully understand the molecular biology of an organism. In general, it is difficult to identify previously uncharacterised cis-acting elements with an unknown consensus sequence. The task is especially problematic with viruses containing regions of limited or no similarity to other previously characterised sequences. Fortunately, the fast increase in the number of sequenced genomes allows us to detect some of these elusive cis-elements. In this work, we introduce a web-based tool called cRegions. It was developed to identify regions within a protein-coding sequence where the conservation in the amino acid sequence is caused by the conservation in the nucleotide sequence. The cRegion can be the first step in discovering novel cis-acting sequences from diverged protein-coding genes. The results can be used as a basis for future experimental analysis. We applied cRegions on the non-structural and structural polyproteins of alphaviruses as an example and successfully detected all known cis-acting elements. In this publication and in previous work, we have shown that cRegions is able to detect a wide variety of functional elements in DNA and RNA viruses. These functional elements include splice sites, stem-loops, overlapping reading frames, internal promoters, ribosome frameshifting signals and other embedded elements with yet unknown function. The cRegions web tool is available at http://bioinfo.ut.ee/cRegions/.

## Introduction

Mostly, the amino acid sequence of a protein is conserved in order to maintain its function and structure. However, the conservation may also be caused by the selection at the nucleic acid level due to essential cis-acting sequences located in the protein-coding region. Thus, certain regions in a protein-coding sequence might encode specific amino acids not because of the selective pressure to the amino acid sequence, but because of the conservation at the nucleic acid level in DNA or RNA. There can be multiple reasons: the existence of nucleic acid secondary structures, splice sites, binding sites for proteins (e.g. transcription factors) or short RNAs, internal promoters, ribosome frameshifting signals, subgenomic promoters in RNA viruses, viral packaging signals and other regulatory elements. Additionally, the conservation at nucleic acid level might exist due to overlapping reading frames, which are common in viruses but also occur in cellular organisms (Okazaki et al., 2002; Shendure & Church, 2002; Veeramachaneni et al., 2004; Belshaw, Pybus & Rambaut, 2007; Rancurel et al., 2009; Chirico, Vianelli & Belshaw, 2010; Firth, 2014).

Computational annotation is extremely important when the experimental annotation is impracticable, for example, in case of organism or hosts which are uncultivable. However, thanks to the massive deployment of second-generation sequencing, the number of complete or near-complete genomes of previously unknown viruses has increased tremendously. This is one of the reasons why comparative analysis and computational annotation is needed to get some insight into the molecular biology of these viruses. Additionally, it has been shown that a large number of these new viruses will most likely constitute a new viral genus or even a family (Labonté & Suttle, 2013; Zhou et al., 2013; Dutilh et al., 2014; Rosario et al., 2015; Yutin, Kapitonov & Koonin, 2015; Zhang et al., 2015; Dayaram et al., 2016; Krupovic et al., 2016; Simmonds et al., 2017). Sometimes these novel viral species are too different from the existing species in the database; therefore, the homology-based methods are unable to detect any similarities to previously characterised sequences or cis-elements. However, thanks to the current advances in sequencing, the number of different relatives of the same virus can be quite high. Thus, a lot of evolutionary information is available. Proper analysis of these sequences can uncover at least some of the embedded functional elements and give us a better understanding of a virus (Gog et al., 2007; Firth, 2014; Sealfon et al., 2015).

Several studies have used synonymous substitution restriction to identify overlapping or embedded functional elements in coding sequences of viruses (Simmonds & Smith, 1999; Gog et al., 2007; Mayrose et al., 2013; Firth, 2014; Sealfon et al., 2015). However, most of these methods detect overlapping or embedded elements only at a low resolution (over several codons) and often lack available implementation and/or a web interface. Here, we introduce the cRegions, which identifies regions within diverged protein-coding sequences where the distribution of observed nucleotides is significantly different from the expected distribution which is based on the amino acid composition and codon usage. Therefore, cRegions does not identify regions of excess synonymous constraint strictly, but rather compares observed codon usage to predicted codon usage at a single-nucleotide resolution. This allows cRegions to identify potential embedded functional cis-elements in coding sequences regardless of their nature. To demonstrate the capabilities of the cRegions web tool, we used the non-structural and structural polyprotein of alphaviruses as an example.

### Implementation

The overall principle of the cRegions tool is to compare observed nucleotide frequencies to expected probability distribution and calculate appropriate metrics (described below) to detect regions where the coding sequence is more conserved than expected. Scripts used in the cRegions web tool are available in GitHub repository at https://github.com/bioinfo-ut/cRegions. The workflow of cRegions is as follows:

1. Two inputs are required: a protein multiple sequence alignment (MSA) and nucleic acid sequences containing coding sequences (CDS) of respective proteins. Both inputs have to be in FASTA format. mRNA or the full genome can be used instead of the exact CDS. However, the coding sequence must not contain introns.
2. Protein alignment is converted into a corresponding codon alignment using respective coding sequences with PAL2NAL (Suyama, Torrents & Bork, 2006).
3. Henikoff position-based sequence weights are calculated using the codon alignment (Henikoff & Henikoff, 1994).
4. Codon usage bias is calculated from the codon alignment. Calculated proportions are adjusted by Henikoff position-based sequence weights to account for non-uniform phylogenetic coverage (Fig. S1). Codon preference for serine in the TCN block and in the AG[A/G] are calculated separately.
5. Expected nucleotide proportions are calculated for each position in the codon alignment based on the amino acid sequence and the codon usage bias (calculated or provided by the user). Henikoff position-based sequence weights are used to adjust expected proportions (Fig. S2).
6. The observed nucleotide frequencies are compared to the expected probability distribution of nucleotides in each position. The comparison is made only for positions with amino acids having more than one codon. Three different metrics are used for this:
  - The algorithm uses R (R Development Core Team, 2014) to calculate *p*-values of Chi-square goodness of fit test (chisq.test) for each column in the codon alignment. The test allows us to see whether the observed distribution of nucleotides is significantly different from expected distribution. We use the negative logarithm of the *p*-value of the Chi-square goodness of fit test as the metric. Bonferroni correction is used to show the threshold with significance level α = 0.05.
    $$p{\rm{ - value}} = {\rm{chisq}}.{\rm{test}}({\rm{c}}({{\rm{A}}_{{\rm{obs}}}},{{\rm{C}}_{{\rm{obs}}}},{{\rm{G}}_{{\rm{obs}}}},{{\rm{T}}_{{\rm{obs}}}}),\quad {\rm{p}} = {\rm{c}}({{\rm{A}}_{{\rm{exp}}}},{{\rm{C}}_{{\rm{exp}}}},{{\rm{G}}_{{\rm{exp}}}},{{\rm{T}}_{{\rm{exp}}}}))$$
    The subscript ‘obs’ indicates observed frequencies, the subscript ‘exp’ indicates expected proportions.
  - The second metric is the root-mean-square deviation (RMSD). Only nucleotides which have a predicted probability over zero are included in the RMSD calculation.
    $${\rm{RMSD}} = \sqrt {{1 \over 4}\left[ {{{\left( {{A_{{\rm{obs}}}} - {A_{{\rm{exp}}}}} \right)}^2} + {{\left( {{C_{{\rm{obs}}}} - {C_{{\rm{exp}}}}} \right)}^2} + {{\left( {{G_{{\rm{obs}}}} - {G_{{\rm{exp}}}}} \right)}^2} + {{\left( {{T_{{\rm{obs}}}} - {T_{{\rm{exp}}}}} \right)}^2}} \right]}$$
    The subscript ‘exp’ indicates expected frequencies.
  - The third metric is the maximum difference (MAXDIF), which selects only a single nucleotide from each column having the highest absolute difference between predicted and observed values.
    $${\rm{MAXDIF}} = \max \left( {\left| {{A_{{\rm{obs}}}} - {A_{{\rm{exp}}}}\left| , \right|{C_{{\rm{obs}}}} - {C_{{\rm{exp}}}}\left| , \right|{G_{{\rm{obs}}}} - {G_{{\rm{exp}}}}\left| , \right|{T_{{\rm{obs}}}} - {T_{{\rm{exp}}}}} \right|} \right)$$
    The subscript ‘exp’ indicates expected frequencies.

In all cases, the larger numerical value of a metric indicates higher conservation at the nucleic acid level. Additionally, if a position in the codon alignment has more than 20% of gaps, the metric is not calculated for that position (Fig. S3).

## Material and Methods

### Alphavirus dataset

In the present work, we used the non-structural and structural polyprotein of alphaviruses as an example. Sequences were downloaded from the NCBI viral genome database (non-redundant dataset, 16 April 2018). The first dataset consists of 24 known alphaviruses (Table S1). The non-structural polyprotein dataset was further divided into two sub-datasets. The first subset (SFV dataset) consists of seven viruses from genus Alphavirus, all belonging to the ‘SFV Complex’ (Table S1) (Forrester et al., 2012). The second subset was formed by nine ‘New World’ alphaviruses (Table S1).

### cRegions and synplot2

Protein sequences were aligned with MAFFT (Katoh et al., 2002) using the default settings at http://www.ebi.ac.uk/Tools/msa/mafft/. Graphs were created with default settings using a sliding window with size one unless stated otherwise. Codon alignments for the synplot2 (Firth, 2014) were created with PAL2NAL (Suyama, Torrents & Bork, 2006), thus the input alignments for synplot2 and cRegions are identical. In this study the smallest possible window size was used for synplot2, giving the resolution of three codons (2*n* + 1 codons). In case of synplot2, significant hits were selected at threshold *p* < 10^−5^. It is less conservative compared to the threshold used in the synplot2 paper (Firth, 2014).

### Sequence weighting

Henikoff position-based sequence weights are used to compensate for the over-representation of well-sequenced taxa in the MSA (Henikoff & Henikoff, 1994). Contrary to the original work of Henikoffs, we applied position-based weights to nucleotide sequences in the codon alignment, not to protein sequences. Thus, including variance at the codon level. Predicted nucleotide proportions for each position in the codon alignment are adjusted with sequence weights.

### Sliding window mode

Cis-acting elements may be longer than a single codon, for example, dual-coding regions, thus the possibility to calculate a single metric over consecutive codons may be preferred. The cRegions web tool allows the user to set the window size from one to 1/9 of the length of the codon alignment. By default, the third codon position is used in the sliding window mode, as it is most informative. An additional threshold exists in the sliding window mode. The threshold is for skipping columns instead of terminating the metric calculation for these consecutive positions. For example, if there is an insertion in a single sequence, the position should be skipped and the next codon included in the current window instead. It should be noted that skipping can happen several times in a row. By default, if a position in an MSA has more than 90% of gaps, it is skipped in the sliding window mode. It should be noted that the threshold for skipping gaps and the threshold for metric calculation are different parameters (Fig. S3). In case of RMSD and MAXDIF, an arithmetic mean is calculated over consecutive codons. However, a *p*-value of Chi-square test is calculated based on observed values that are added over all consecutive codons. Again, Bonferroni correction is used to show the threshold with significance level α = 0.05. It should be kept in mind that adjacent positions with low metric values will decrease the value of a single conserved position if the window size is larger than one.

### Visualisation

The cRegions web tool uses ‘highcharts’ libraries to visualise results (http://www.highcharts.com/). Alignment visualisation is provided by MSAViewer (Yachdav et al., 2016). The combination of highcharts and MSAViewer allows the user to pinpoint (by clicking on the point) and navigate directly to a conserved region or nucleotide. In addition to scatter plots of different metrics, cRegions web tool displays an interactive graph of codon usage. Codon usage is calculated over all analysed sequences. A table of codon frequencies and a file with tab-separated values are included in the downloadable zip container. The same codon table can be used as an input for the cRegions algorithm.

### VEEV sequences

The Venezuelan equine encephalitis virus (VEEV) neighbour sequences were downloaded from the NCBI viral genomes database (https://www.ncbi.nlm.nih.gov/genomes/GenomesGroup.cgi?taxid=11018, 18 May 2018). A total of 11 entries were removed as they did not have annotated full-length non-structural polyprotein. Identical protein sequences were removed using jalview ‘remove redundancy 100’ (Waterhouse et al., 2009). The final dataset contained a total of 94 isolates, including 93 VEEV neighbour sequences and a reference VEEV sequence (NC_001449).

### Randomly mutated protein-coding sequences

A random 3,000 nt long protein-coding sequence was created with SMS v2 tool (http://www.bioinformatics.org/sms2/random_coding_dna.html). A different number of random mutations (25–1,600) were introduced into that sequence with the SMS2 mutate tool (http://www.bioinformatics.org/sms2/mutate_dna.html) (Stothard, 2000). In total, we generated 3 × 8 datasets. Each set consisted of one original randomly generated protein-coding sequence and six, nine or 14 randomly mutated sequences with a different number of mutations per bp. For the set with 15 sequences we generated different initial protein-coding sequence to remove a bias which could occur if we only use one protein-coding sequence as a seed. These sequences do not need aligning, because homologous nucleotides are already aligned.

### Threshold correction for nearly identical sequences

cRegion algorithm assumes that, in general, the distribution of nucleotides at each position in the MSA correspond to an average codon usage of the same sequences under analysis. The assumption is reasonable if the sequences under analysis have diverged. However, in the case of nearly identical sequences, the distribution of nucleotides in each position in the MSA is more similar to observed nucleotide proportions rather than to average codon usage. Therefore, the expected proportions are more similar to observed proportions. Thus, even if using Bonferroni correction, the Chi-square test may still give many potentially false positive signals. Therefore, we need to adjust the threshold such a way that in the case of nearly identical sequences the threshold is stricter. For that, we also include the average pairwise identity of nucleotide sequences into calculations of expected nucleotide proportions at each position. The expected nucleotide proportions are adjusted by the observed proportions which depend on the average pairwise identity of nucleotide sequences. The exponent value was found empirically.

$${\left( {{A_{{\rm{exp}}}},{C_{{\rm{exp}}}},{G_{{\rm{exp}}}},{T_{{\rm{exp}}}}} \right)_{{\rm{adj}}}} = \left( {{A_{{\rm{exp}}}},{C_{{\rm{exp}}}},{G_{{\rm{exp}}}},{T_{{\rm{exp}}}}} \right) + {i_n}\hskip-3.7pt^7*\left( {{A_{{\rm{obs}}}},{C_{{\rm{obs}}}},{G_{{\rm{obs}}}},{T_{{\rm{obs}}}}} \right)$$

*i_n_* = the average pairwise identity of nucleotide sequences

In addition to the previous adjustment of expected values, also the threshold itself is adjusted. The threshold correction depends on the ratio between the average pairwise identity of nucleotide and protein sequences and the number of sequences in the MSA.

$${{t_{{\rm{corrected}}}} = {t_{{\rm{current}}}}*{{{i_n}} \over {{i_p}}}*\left({1 + {i_n}\hskip-3.7pt^d} \right)}$$

*t* = threshold − log10(*p*–value)
*i_n_* = the average pairwise identity of nucleotide sequences
*i_p_* = the average pairwise identity of protein sequences
*d* = the number of sequences in the multiple sequence alignment

## Results

### Alphaviruses

**Figure 1:**
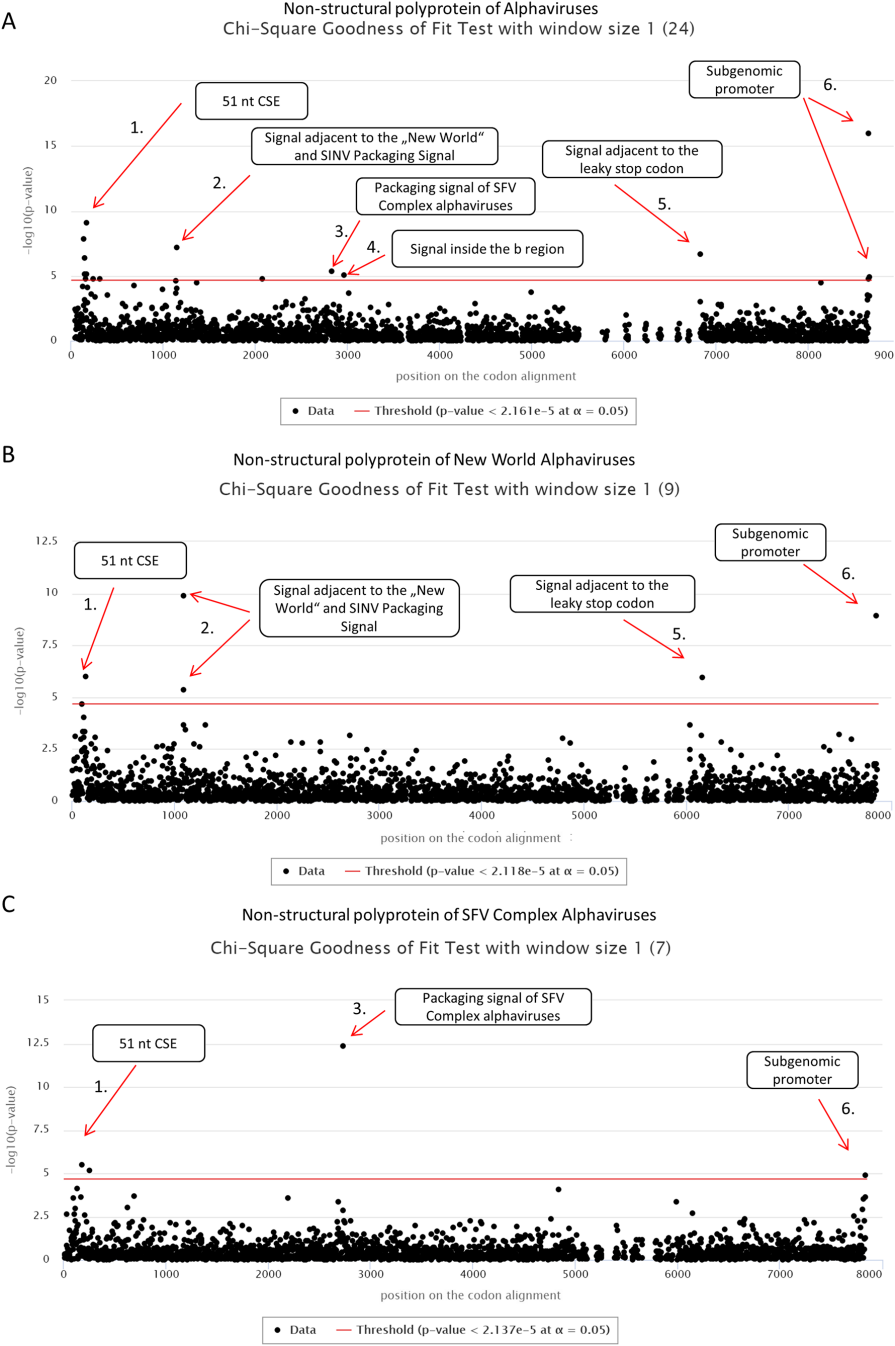
cRegions analysis of non-structural polyproteins of alphaviruses using Chi-square goodness of fit test. On each graph, the *y*-axis shows the negative logarithm of the Chi-square goodness of fit test *p*-value and the *x*-axis shows the position in the codon alignment. The red line represents the significance threshold (α = 0.05 with Bonferroni correction). (A) Non-structural polyprotein alignment of all 24 Alphaviruses. (B) Non-structural polyproteins of ‘New World’ Alphaviruses. (C) Non-structural polyproteins of ‘SFV Complex’ Alphaviruses. Non-structural polyprotein sequences were aligned with MAFFT version 7 using the default settings at http://www.ebi.ac.uk/Tools/msa/mafft/ (Katoh et al., 2002). Graphs were generated with sliding window mode (window size = 1). On the panel title, the number of analysed sequences is shown in parentheses.

First, we applied cRegions and synplot2 on all 24 non-structural polyproteins of alphaviruses (see also ‘alphavirus dataset’ example on the cRegions homepage). We detected a total of six significant signals with cRegions (Fig. 1A) and three significant signals with the synplot2 (Figs. S4A and S5A). The first signal from the 5′ end was recognised by both programs (Fig. 1A) and spanned from positions 138 to 174 in the codon alignment (Table 1). It is a conserved sequence element (CSE) called ‘51 nt CSE’, which acts as an enhancer for the RNA synthesis, affecting viral replication. This CSE forms two stem-loops and is located at positions 155–205 in the Sindbis virus (SINV) genome (Niesters & Strauss, 1990). Thus, the detected signal lies exactly in the region (Table 1).

**Table 1:** Detected signals in the codon alignment in different datasets and respective positions in SFV and SINV genome.

	Signal	Description	Dataset	Position on the codon alignment	SFV*	SINV*
Non-structural polyprotein	1	51 nt CSE	All (Fig. 1A)	138–174	184–220	161–197
	2	Signal adjacent to capsid binding region	All (Fig. 1A)	1,149	NA	1,148
			New world (Fig. 1B)	1,086 and 1,092	NA	1,142 and 1,148
	3	Packaging signal of SFV Complex alphaviruses	All (Fig. 1A)	2,835	2,812	2,804
			SFV Complex (Fig. 1C)	2,730	2,812	2,804
	4	Signal inside the b region	All (Fig. 1A)	2,967	2,944	2,936
	5	Signal adjacent to leaky stop codon	All (Fig. 1A)	6,834	5,536	5,768
			New world (Fig. 1B)	6,159	NA	5,888
	6	Subgenomic promoter of alphaviruses	All (Fig. 1A)	8,658 and 8,664	7,354 and 7,360	7,583 and 7,589
Structural polyprotein	1	UUUUUUA motif	All (Fig. 2)	2,673–2,679	9,825–9,831	10,022–10,028

**Notes:**

NA, not applicable.

* Shows respective position(s) on the SFV (NC_003215) and SINV (NC_001547) genome.

The second significant hit, a single nucleotide at position 1,149, was detected only by the cRegions algorithm. However, two adjacent positions 1,143 and 1,146 were just below the threshold. In the New World alphavirus dataset, in addition to position 1,149, 1,143 was also significant. The signal is just adjacent to the packaging signal of SINV and New World alphaviruses (see also ‘New World alphavirus dataset’ example on the cRegions the homepage). It has been shown that a 570 nt fragment positions 684–1,253 from the SINV binds to the viral capsid protein and is required for packaging of SINV. The detected signal lies in this region (Weiss et al., 1989). However, when we analysed VEEVs separately we were able to detect the positions of phylogenetically conserved predicted stem-loops (Fig. S7). The results are similar to the work done by Kim et al. (2011).

The third and the fourth signal are also single nucleotides at positions 2,835 and 2,967, respectively (Fig. 1A). Both signals are located inside nsp2 conserved region called region b. This 266-nucleotide region is located from 2,726 to 2,991 in the SFV genome (White, Thomson & Dimmock, 1998). Previous deletion mutation analysis has shown that nucleotides from 2,767 to 2,824 in the b region are required for efficient packaging of SFV genome. (White, Thomson & Dimmock, 1998). The first signal is located in that region. Additionally, analysis of ‘SFV Complex’ viruses separately led to increased significance of the first signal (Fig. 1C) and the same signal became visible with synplot2 (Figs. S4B and S5B). Expectedly, both signals disappeared in the New World dataset, as the packaging signal is in a different location in these viruses (Fig. 1B). Therefore, dividing datasets to different subsets may help to detect signals that are only characteristic to smaller subgroups.

The fifth significant hit is a single nucleotide at position 6,834. It is downstream of the ‘leaky’ stop codon (stop codon is at 6,814–6,816 on the codon alignment and in the SINV genome at nt 5,748–5,750). Synplot2 was able to detect a much larger region compared to cRegions in the same area (Figs. S4A and S5A). The detected signal is a 3′ stem-loop RNA secondary structure immediately adjacent to the stop codon (+13 nt downstream of the stop codon in SINV). For many alphaviruses, including VEEV and SINV, it has been reported to influence read-through. In the SINV genome, the double helix part (the stem) of the stem-loop is predicted to form between the two regions: 5,763–5,772 and 5,928–5,939 (Firth et al., 2011). Therefore, the detected signal at position 6,834 (5,768 in SINV) is inside the first region. However, when we analysed VEEVs separately, we were able to detect multiple significant signals inside this stem-loop region (Fig. S7).

The sixth signal consists of two positions 8,658 and 8,664 on the codon alignment (Fig. 1A; Figs. S4A and S5A). The signal is located within the subgenomic promoter of alphaviruses (Raju & Huang, 1991; Rupp et al., 2015).

**Figure 2:**
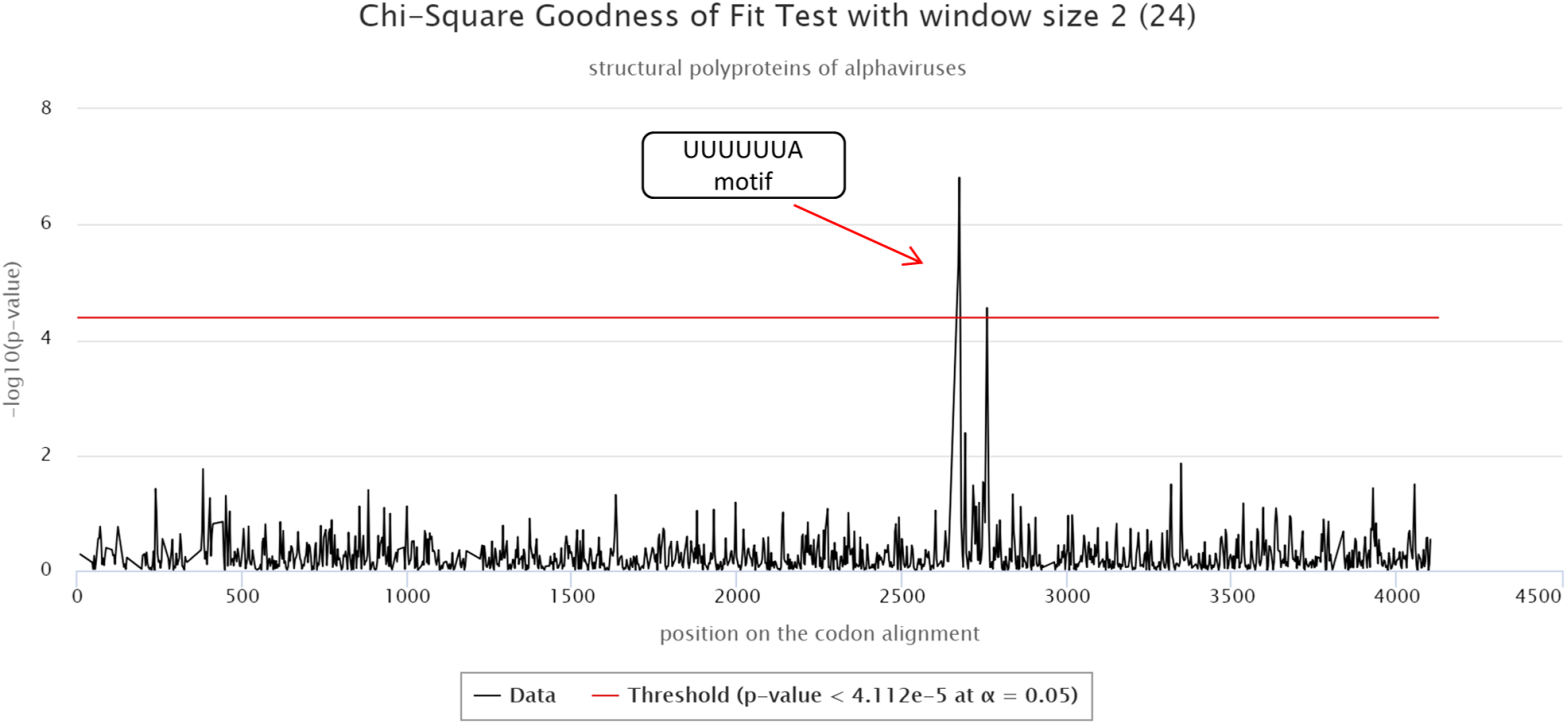
cRegions analysis of structural polyproteins of alphaviruses. A significant signal was detected in codon alignment positions 2,643–2,649, the region corresponds to a known UUUUUUA motif. The *y*-axis on the plot shows the negative logarithm of the Chi-square goodness of fit test *p*-value and the *x*-axis shows the position on the codon alignment. The red line represents the significance threshold (α = 0.05 with Bonferroni correction). Structural polyprotein sequences were aligned with MAFFT version 7 using the default settings at http://www.ebi.ac.uk/Tools/msa/mafft/ (Katoh et al., 2002). Sliding window size 2 was used.

The cRegions and the synplot2 were also applied to the structural polyproteins of alphaviruses (see also ‘alphavirus structural dataset’ example on the cRegions homepage). Sliding window size 2 was used with cRegions. A strong signal was detected in positions 2,643–2,649 on the codon alignment, which corresponds to a UUUUUUA motif (Fig. 2). The motif is responsible for a frameshift in a structural protein (Firth et al., 2008; Chung, Firth & Atkins, 2010). The same signal was detected with the synplot2 (Fig. S6).

### Requirements on sequences

**Figure 3:**
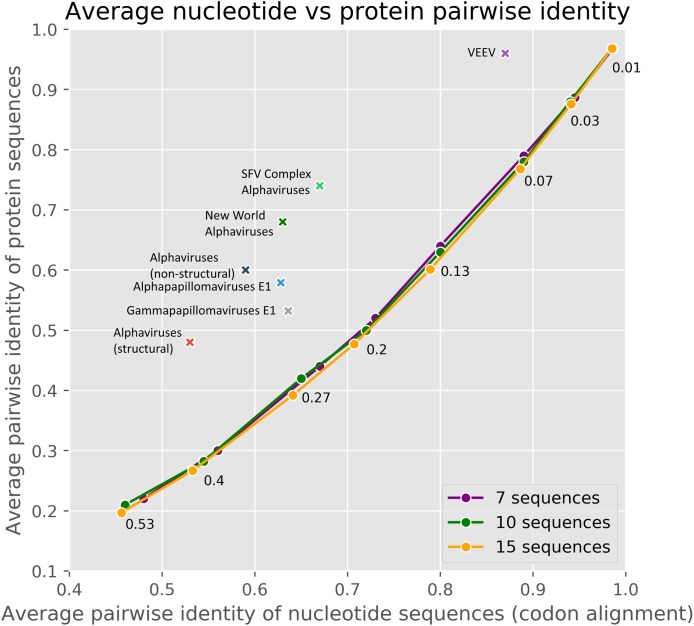
The average pairwise identity of nucleotide sequences from codon alignment plotted against the average pairwise identity of protein sequences in respective MSA. A different number of random mutations (25–1,600) were introduced into a randomly generated 3,000 nt long protein-coding sequence with the SMS2 mutate tool. On the plot, three different lines represent sets of 7, 10 or 15 sequences. Therefore, each data point on a line consisted of one original protein-coding sequence and 6, 9 or 14 mutated sequences. The number near the point shows mutations per base pair. The real datasets are marked with crosses. Majority of real datasets visualised on the plot are from this paper, others originate from our previous paper (Puustusmaa & Abroi, 2016).

The method used in cRegions has some limitations and prerequisites (Puustusmaa & Abroi, 2016). First, the sequences under study must have diverged. Second, the embedded functional element must have been under selection. To help users to evaluate their sequences in these aspects, we added an interactive version of Fig. 3 to the web tool. The plot visualises the sequences under study in comparison to randomly mutated sequences and sequences thoroughly analysed in the previous or current study with respect to divergence and selection. To evaluate divergence and selection we used the relationship between average pairwise nucleotide identity and average pairwise amino acid identity (Fig. 3). As shown in Fig. 3, randomly mutated simulated sequences form a clear and narrow assembly on the plot. Randomly mutated sequences with a defined extent (N mutations per bp) were used to model neutral evolution and/or non-diverged sequences (more details in ‘Materials and Methods’). The naturally occurring sequences used in the previous and in the current study locate clearly away of the simulated sequences.

### Sequences having low divergence or/and having close to neutral evolution

**Figure 4:**
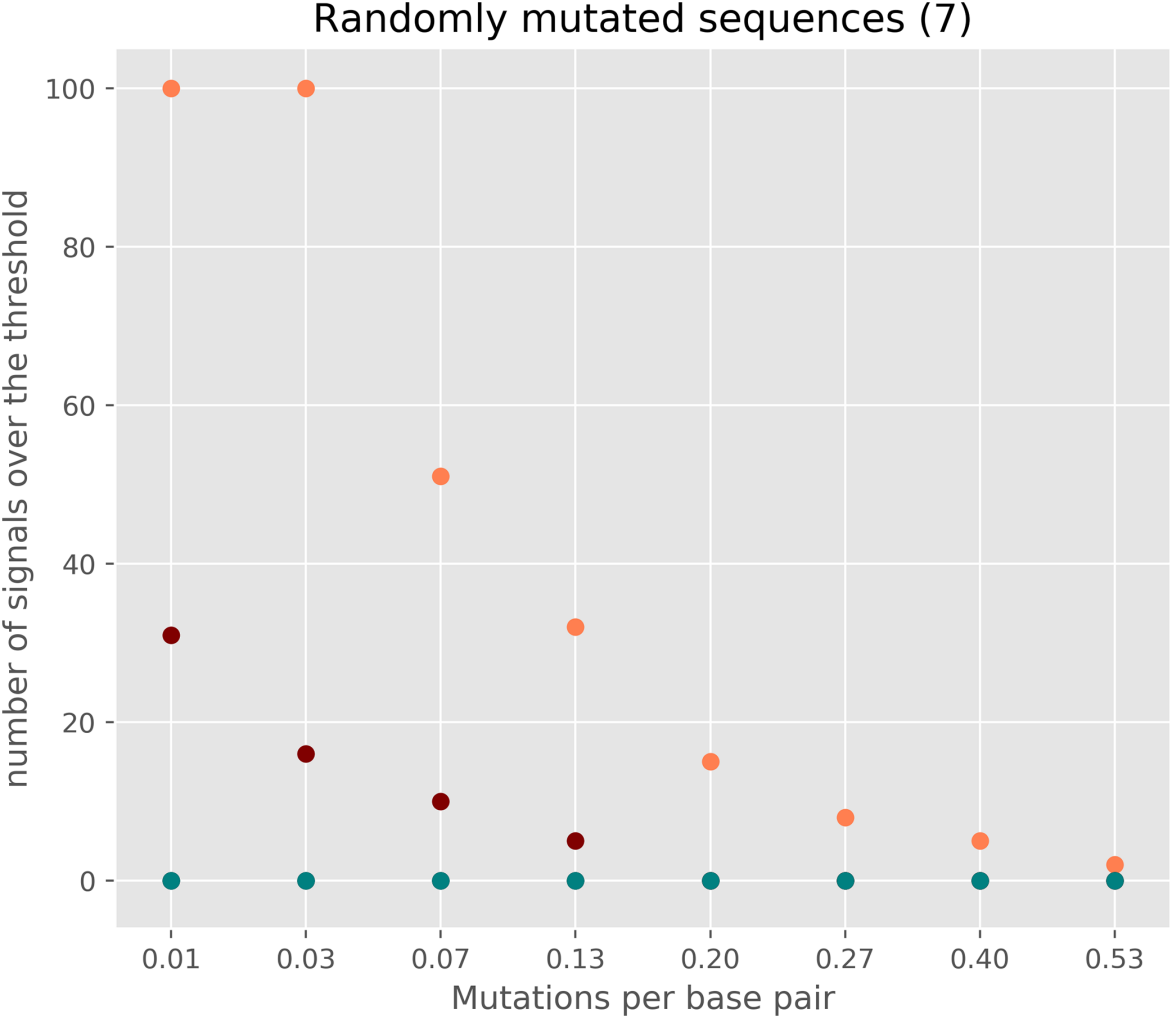
The number of signals in randomly mutated sequences. A random 3,000 nt long protein-coding sequence was created with SMS v2 tool (http://www.bioinformatics.org/sms2/random_coding_dna.html) (Stothard, 2000). A different number of random mutations (25–2,000) were introduced into the original randomly generated protein-coding sequence six times with the SMS v2 mutate tool (http://www.bioinformatics.org/sms2/mutate_dna.html) (Stothard, 2000). Each simulated dataset consisted of seven sequences each having the same number of mutations. Orange circles show the number of signals exceeding the threshold without a threshold correction and using window size 1. Red circles show the number of signals with sliding window size 2. The deep blue-green (teal) shows the number of signals exceeding the threshold when applying threshold correction.

As cRegions was designed to work on diverged sequences, the method may give potential false positive signals in low divergence sequences or in sequences locating close to neutrally evolving sequences (Fig. 4; Fig. S8). To avoid this, we recommend enabling threshold correction on the web tool. By enabling this option expected values are corrected with observed values and the adjusted threshold is calculated (see ‘Materials and Methods’). This removes most of the false positive signal from sequences that are close to randomly mutating sequences (Fig. 4; Fig. S8). We would like to note that the correction is needed only in the case of sequences which are close to the neutrally/randomly evolving sequences (Fig. 3). Another option is to use synplot2 which uses neutral evolution as its null hypothesis (Firth, 2014)

## Discussion

Given sufficient evolutionary time, the conservation of amino acid residues in different homologous sequences will not necessarily imply the same conservation at nucleotide level due to the redundancy of the genetic code. However, it can be reasoned that functionally important cis-acting elements embedded in protein-coding sequences will be evolutionarily conserved, even if these regions are subject to constant evolutionary pressure both through their translation product (amino acid sequence) and cis-acting functions.

Previous studies have produced numerous valuable methods for detecting overlapping or embedded functional elements in coding sequences, mainly by identifying regions of excess synonymous constraints (Simmonds & Smith, 1999; Gog et al., 2007; Mayrose et al., 2013; Firth, 2014; Sealfon et al., 2015). Currently, only Firth has created a web interface to the synplot2 and Sealfon et al. have implemented FRESCo as a usable batch script. However, running a script in a Unix terminal might be a daunting task for a biologist, therefore, a web interface is essential for bioinformatics tools to be widely adopted. Additionally, FRESCo needs a phylogenetic tree as an input, which depends highly on the construction method (Sealfon et al., 2015).

During evolution, homologous protein-coding sequences accumulate random substitutions by switching to different synonymous and non-synonymous codons. In addition, if purifying selection acts on a protein sequence, synonymous substitutions are favoured over non-synonymous substitutions. However, synonymous codons are not used with equal proportions (Moriyama & Powell, 1997; Duret, 2000, 2002; Castillo-Davis & Hartl, 2002; Zhao, Liu & Frazer, 2003; Plotkin et al., 2006; Bahir et al., 2009; Camiolo, Farina & Porceddu, 2012; Villanueva, Martí-Solano & Fillat, 2016). One hypothesis that explains the preferential use of synonymous codons is the adaptation towards translational efficiency and gene expression (Moriyama & Powell, 1997; Duret, 2000, 2002; Castillo-Davis & Hartl, 2002; Plotkin et al., 2006; Camiolo, Farina & Porceddu, 2012; Chaney et al., 2017). Gene expression and translational efficiency can also be affected by consistent under- or over-representation of certain codon pairs. Multiple works conducted on RNA viruses have shown that altering codon pair frequencies towards those that are disfavoured in their host will reduce virus replication (Coleman et al., 2008; Martrus et al., 2013; Le Nouen et al., 2014). However, the effect may be an artefact of changes in the CpG and UpA dinucleotide frequencies instead (Tulloch et al., 2014).

In addition, host adaptation theory (tissue adaptation) may explain the preferential use of synonymous codons. It has been shown that the codon usage is strongly related to the specific host in both bacterial and human viruses. Also, the highest level of adaptation to host codon usage is for proteins which appear abundantly in the virion, meaning that the codon usage of virion and non-virion proteins differ (Bahir et al., 2009). Additionally, Villanueva, Martí-Solano & Fillat (2016) have proved that there is a difference in the codon usage between the host-interacting protein and the rest of structural late phase proteins. Thus, it can be reasoned that each protein-coding gene may have different preferential use of synonymous codons. Therefore, incorporating the preferential use of synonymous codons of the same set of genes into the model is reasonable.

However, authors of the synplot2 used neutral evolution as the null model, therefore not including codon usage bias into the analysis. They reasoned that it was not required based on the results and it would be impossible to accurately estimate given the limited genome size of RNA viruses (Firth, 2014). Also, Sealfon et al. (2015) argued that the genetic region of a typical virus is only about a thousand of codons long, therefore, there may be insufficient information to characterise the codon usage bias. To date, only the method published by Gog et al. (2007) incorporated codon usage bias into their model.

Calculating the preferential use of synonymous codons may result in incorrect assessment on many occasions. First, if the analysis is performed on sequences with low divergence, then the estimation of codon usage may be biased. Second, a large overlapping region or the abundance of rare codons in a gene may also affect codon usage estimation. The same conclusion was reached in the synplot2 paper by Firth, where he noted that the divergence parameters of the null model are determined from the full coding region and if the alignment contains extensive overlapping regions then the neutral divergence rates will be underestimated (Firth, 2014). As a remedy, cRegions web tool allows the user to input a custom codon table, which may have been calculated from a larger set of genes and be more suitable in some cases. Additionally, cRegions allows the user to choose between 17 different codon tables that are also implemented in PAL2NAL (Suyama, Torrents & Bork, 2006).

However, not all synonymous codons should be treated as equals. In case of serine, two mutations are necessary to move from AG[A/G] serine codon block to TCN codon block (in the standard codon table). Therefore, usage for codons in the TCN block and in the AG[A/G] are calculated separately. For example, if only AG[A/G] serine was observed, then only AG[A/G] codon proportions were used for predictions and vice versa. This is not implemented in the method published by Gog et al. (2007). Also, it was not implemented in the original publication of the method (Puustusmaa & Abroi, 2016).

As it is impossible to assess the conservation at the nucleic acid level if an amino acid is encoded only by a single codon (e.g. methionine and tryptophan), therefore these amino acids are excluded from the analysis. For these positions, it is unknown if the conservation is due to an amino acid or DNA/RNA constraint or by pure chance. cRegions will not calculate metrics for these positions. In the work done by Gog et al. (2007) these positions were masked to ensure that they were not flagged as low scoring and included them into the moving average.

Databases often contain a redundant set of sequences. Therefore, it is very important to include phylogenetic weighting in the analysis. A redundant and biased dataset or a dataset with low variability will affect predictions and therefore may cause false positive signals. Henikoff position-based sequence weighting was used to compensate for the over-representation of similar sequences or taxa in the codon alignment (Henikoff & Henikoff, 1994). Tree-based weighting methods were excluded due to the uncertain root location, which may give lower weights to sequences close to the root, causing distantly related sequences to be down-weighted. The cRegions algorithm calculates weights for each sequence in the codon alignment, thus including variance at the nucleic acid level. Weighting was not implemented in the original publication of the method (Puustusmaa & Abroi, 2016). Synplot2 also uses sequence weighting. However, it should be noted, phylogenetic weighting only affects the results if the initial set of sequences was biased.

Cis-acting elements may be longer than a single codon, for example, dual-coding regions, thus the possibility to calculate a single metric over consecutive codons may be preferred. Sliding window approach is used in synplot2, FRESCo, and method described by Gog et al. (2007). The method used in Gog et al. (2007) calculated MDP score over a sliding window of 10 codons and minimum window size in the synplot2 web interface is three at (*n* = 1). However, cis-acting sequences shorter than three codons (e.g. canonical splice acceptor site in Mammalia CAG|G) may be masked by adjacent low scoring areas. Thus, in contrast to them, cRegions allows also single-codon resolution. We would like to note that synplot2 provides single-codon scores in an output text file and these can be used for analysis of regions shorter than three codons.

In this study, we applied cRegions to the non-structural and structural polyprotein of alphaviruses as an example. The final dataset contained 24 sequences (see Materials and Methods). The diversity and the number of sequences were sufficient in our analysis to detect the majority of known cis-acting elements in alphaviruses.

Several alphaviruses contain an in-frame termination codon and use termination read-through to produce the p1234 non-structural polyprotein (Strauss, Rice & Strauss, 1983; Li & Rice, 1989; Myles et al., 2006). Dataset used in this work includes nine cases of known termination read-throughs: WHAV, AURAV, EILV, BEBV, NDUV, BFV, MADV, VEEV and SESV. However, ONNV, CHIKV, SFV, SPDV and SDV do not have a nonsense codon (at least in reference genomes). Inconsistency between the protein and nucleotide sequences will display warnings, although, the calculation will not be terminated. The presence of a 3′ stem-loop RNA secondary structure immediately adjacent to the stop codon has been reported to influence read-through (Firth et al., 2011). The leaky stop codons in alphaviruses have the next codon CGG or CTA as expected in type II read-through motif. It has been proposed that in most cases of read-through in this class also involve a 3′ RNA structure—often comprising an extended stem-loop structure beginning around eight nt 3′ of the stop codon (Napthine et al., 2012; Firth, 2014). The cRegions tool was able to detect one significant signal inside the double helix part of the stem-loop and one inside the unpaired loop of the stem-loop. However, synplot2 was able to detect a much larger region of the stem-loop, which shows that synplot2 is more suitable in some situations (Fig. 1B; Figs. S4C and S5C).

Both programs: cRegions and synplot2 are able to detect a signal, even if the alignment is not perfect. In the codon alignment of the structural polyprotein, UUUUUUA motif was misaligned in two sequences (SPDV and SDV).

We have shown that cRegions is capable of detecting different cis-elements. However, the method has multiple prerequisites and limitations:

- Protein-coding sequences must have diverged. Thus, sufficient evolutionary time is needed for substitutions to occur in homologous genes in different species/isolates.
- Only those embedded element in a coding-sequence can be detected which are or have been under selection.
- This method is not applicable to neutrally evolving genes. In the case of neutrally evolving genes, we recommend using synplot2 or other similar solutions.
- Cis-acting sequences must be conserved in respect to amino acid sequences.
- It is impossible to assess conservation at the nucleic acid level if an amino acid is encoded by a single codon (e.g. methionine and tryptophan).
- Long dual-coding areas or abundant rare codons will affect codon usage estimation.
- A low number of sequences may reduce the signal to noise ratio.
- Bad alignment quality, especially near large gaps may affect the results. Therefore, usage of different alignment methods or manual correction of the alignment is recommended.

We would like to note that cRegions is not restricted to viral genes, but the method can be applied to any set of diverse set of protein-coding sequence if the prerequisites are fulfilled.

Depending on the number and divergence of the protein and nucleic acid sequences, the size of the region and other conditions, different approaches like the synplot2, FRESCo or the method developed by Gog et al. (2007) may have different sensitivity. Therefore, we advise using different methods side by side to find all putative cis-elements. Also, it should be noted, that any analysis depends on the quality of the input data and even statistically insignificant signals might be biologically very interesting.

## Conclusion

Evolutionary conserved embedded functional elements within an open reading frame are often overlooked as they are difficult to detect without specialised bioinformatics tools (Gog et al., 2007; Sabath, Wagner & Karlin, 2012; Firth, 2014; Sealfon et al., 2015). In this work, we described a web tool called the cRegions. It is built for detecting embedded cis-acting elements from diverged protein-coding sequences. The algorithm behind cRegions compares observed nucleotide (codon) frequencies to preferential use of synonymous codons. Observed and predicted values are compared on three different metrics. The results can be displayed at a single-nucleotide resolution. Our method is able to find different cis-acting elements like splice sites, stem-loops, overlapping reading frames, internal promoters and ribosome frameshifting signals in DNA and RNA viruses.

Web tools like the cRegions and the synplot2 are important for finding functional embedded elements in coding sequences and are easy to use for non-bioinformaticians. The cRegions web tool is available at http://bioinfo.ut.ee/cRegions/ and source code is available in GitHub repository at https://github.com/bioinfo-ut/cRegions.

## Supplemental Information

10.7717/peerj.6176/supp-1 Supplemental Information 1 Alphaviruses dataset. ‘New World’ alphaviruses are marked with asterisk and ‘SFV Complex’ alphaviruses are written in bold.

10.7717/peerj.6176/supp-2 Supplemental Information 2 Codon usage bias is calculated over all positions in all sequences in the codon alignment. (A) Henikoff position-based sequence weights are calculated for each sequence based on the codon alignment. (B) Glutamic acid is encoded by two codons, therefore, the observed proportion for GAA is 0.625 and for GAG 0.375. (C) Henikoff position-based sequence weights are used to compensate for the over-representation of well-sequenced taxa in the multiple sequence alignment. The proportion for GAA and GAG are based on sequence weights.

10.7717/peerj.6176/supp-3 Supplemental Information 3 Predicted nucleotide proportions are adjusted based on sequence weights. (A) Example dataset consists of 7 sequences. Five sequences are very similar; therefore, they have a low weight. Two sequences out of seven are different, therefore, having a high weight. (B) Glutamic acid is encoded by only two codons: GAA and GAG. Only the third position of a codon varies, therefore, including information. The observed proportion for the A nucleotide in the 3rd position of the glutamic acid codon is 0.714 and for G it is 0.286. (C) Predictions based on codon usage give us proportion for A is 0.55 and for G 0.45. By comparing observed and predicted proportions we will get a signal as there is a difference. However, it is a false positive signal due to a biased dataset. (D) Adjusting predictions with sequence weights, we can account for the over-representation of similar sequences. Only nucleotides that were observed are adjusted.

10.7717/peerj.6176/supp-4 Supplemental Information 4 Difference between “allowed gaps” and “skip gaps” parameter. Allowed gaps parameter is a threshold (percentage of gaps in one column) if a metrics (RMSD, MAXDIF, CHISQ) should be calculated to a certain position. Metrics are not calculated at positions (red crosses) where the percentage of gaps in one column exceeds the threshold (percentage of allowed gaps). By default, a position (column) must have less than 20% of gaps. Skip gaps parameter is only used in sliding window mode. It is a threshold for skipping columns during sliding window mode instead of terminating the calculation as in the first three positions (NA). The threshold is used to avoid sliding window calculation termination while encountering insertions in a few sequences. By default, if the proportion of gaps in a position exceeds 90% (in other words, when insertion occurs in less than 10%) then this position is skipped (transparent red column) and next position is included to the window.

10.7717/peerj.6176/supp-5 Supplemental Information 5 Synplot2 analysis of non-structural polyproteins of alphaviruses using 3-codon sliding window (*n* = 1). (A) Non-structural polyprotein alignment of all 24 Alphaviruses in our dataset. (B) Non-structural polyproteins of ‘New world’ Alphaviruses. (C) Non-structural polyproteins of ‘SFV Complex’ Alphaviruses. Non-structural polyprotein sequences were aligned with MAFFT using the default settings at http://www.ebi.ac.uk/Tools/msa/mafft/. Codon alignment was generated with pal2nal (http://www.bork.embl.de/pal2nal/).

10.7717/peerj.6176/supp-6 Supplemental Information 6 Synplot2 analysis of non-structural polyproteins of alphaviruses using 15-codon sliding window (n = 7). Same settings were used in the synplot2 publication (Firth, 2014). (A) Non-structural polyprotein alignment of all 24 Alphaviruses in our dataset. (B) Non-structural polyproteins of ‘New world’ Alphaviruses. (C) Non-structural polyproteins of ‘SFV Complex’ Alphaviruses. Non-structural polyprotein sequences were aligned with MAFFT using the default settings at http://www.ebi.ac.uk/Tools/msa/mafft/. Codon alignment was generated with pal2nal (http://www.bork.embl.de/pal2nal/).

10.7717/peerj.6176/supp-7 Supplemental Information 7 Synplot2 analysis of structural polyproteins of alphaviruses. Significant signal was detected between codons 800–1000, which corresponds to a known UUUUUUA motif. The y-axis on the upper part of the synplot2 figure shows the p-value and the lower part shows obs/exp rato. Structural polyprotein sequences were aligned with MAFFT using the default settings at http://www.ebi.ac.uk/Tools/msa/mafft/. Codon alignment was generated with pal2nal (http://www.bork.embl.de/pal2nal/).

10.7717/peerj.6176/supp-8 Supplemental Information 8 cRegions and Synplot2 analysis of structural polyproteins of 94 VEEV alphaviruses. (A) Two known regions of RNA secondary structures were detected with cRegions using sliding window size 18. (B) Zoomed region of the conserved stem-loops. Locations of the signals in the VEEV genome are also provided (Kim et al., 2014). (C) Zoomed region of the stem-loops adjacent to stop codon. Locations of the signals in the VEEV genome are also provided (Firth et al., 2011). (D) Similar to the work done by Kim et al. sliding window size 25 was used with synplot2 (n = 12). Structural polyprotein sequences were aligned with MAFFT using the default settings at http://www.ebi.ac.uk/Tools/msa/mafft/. Codon alignment was generated with pal2nal (http://www.bork.embl.de/pal2nal/).

10.7717/peerj.6176/supp-9 Supplemental Information 9 The number of signals in randomly mutated sequences. A random 3000nt long protein-coding sequence was created with SMS v2 tool (http://www.bioinformatics.org/sms2/random_coding_dna.html) (Stothard, 2000). A different number of random mutations (25–2000) were introduced into the original randomly generated protein-coding sequence 9 times with the SMS v2 mutate tool (http://www.bioinformatics.org/sms2/mutate_dna.html) (Stothard, 2000). Each simulated dataset consisted of 10 sequences each having the same number of mutations. Orange circles show the number of signals over the threshold without a threshold correction and using window size 1. Red circles show the number of signals with sliding window size 2. The deep blue-green (teal) shows the number of signals over the threshold when applying threshold correction.

## Additional Information and Declarations

### Data Availability

The following information was supplied regarding data availability: GitHub repository: https://github.com/bioinfo-ut/cRegions
