# Supplementary material for: cRegions—a tool for detecting conserved cis-elements in multiple sequence alignment of diverged coding sequences
Source: PeerJ. 2019 Jan 10;6:e6176. doi: 10.7717/peerj.6176 (PMC6330207; doi:10.7717/peerj.6176)
Supplement: Supplemental Information 1 — ‘New World’ alphaviruses are marked with asterisk and ‘SFV Complex’ alphaviruses are written in bold. [file peerj-07-6176-s001.doc]

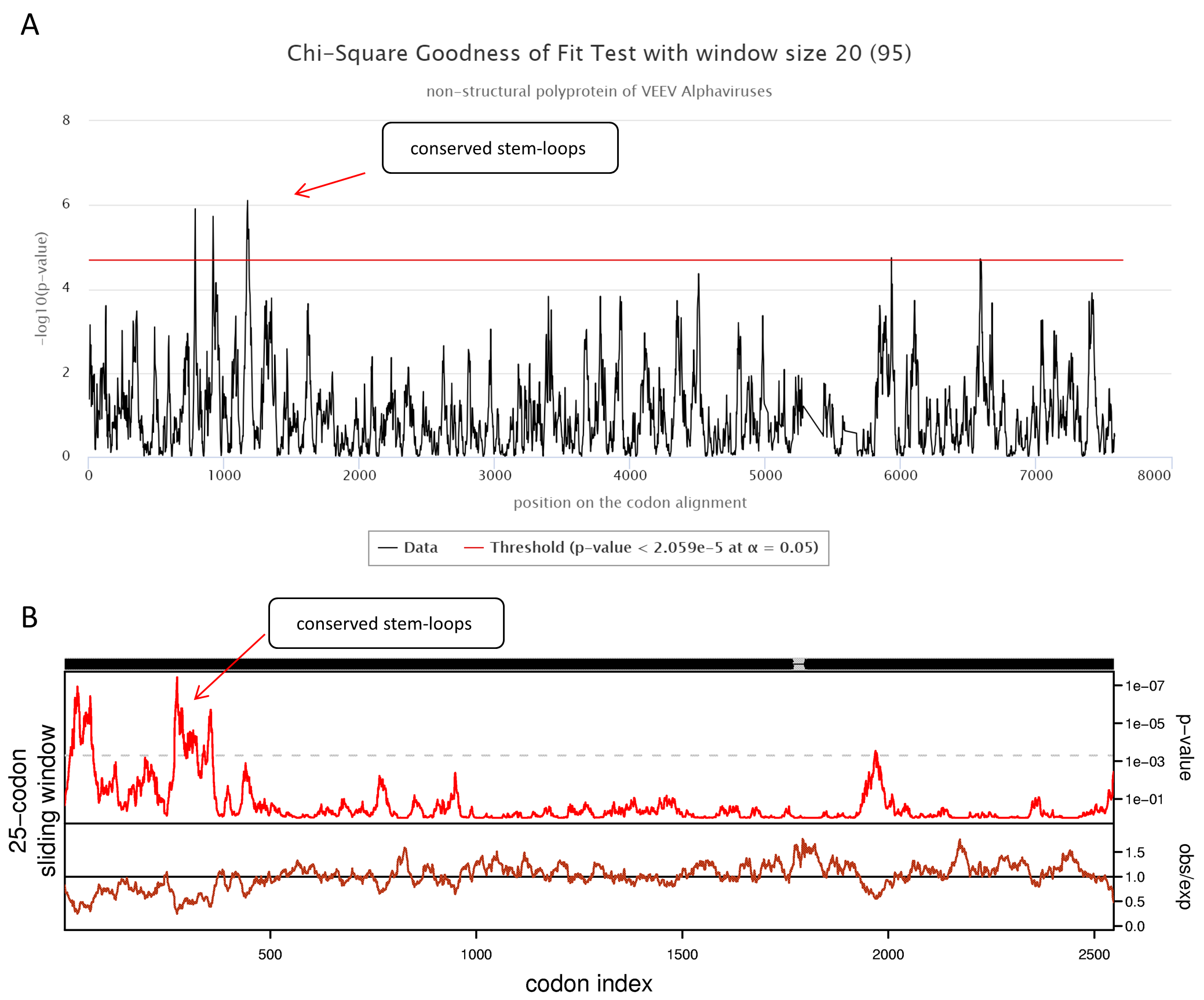


| **Virus Name** | **Abbreviation** | **Taxon ID** | **Accession** |
| --- | --- | --- | --- |
| * Sindbis virus | SINV | 11034 | NC_001547 |
| * Aura virus | AURAV | 44158 | NC_003900 |
| * Eastern equine encephalitis virus | EEEV | 11021 | NC_003899 |
| * Fort Morgan virus | FoMoV | 48544 | NC_013528 |
| * Highlands J virus | HJV | 11024 | NC_012561 |
| * Madariaga virus | MADV | 1440170 | NC_023812 |
| * Venezuelan equine encephalitis virus | VEEV | 11036 | NC_001449 |
| * Western equine encephalomyelitis virus | WEEV | 11039 | NC_003908 |
| * Whataroa virus | WHAV | 48543 | NC_016961 |
| **Bebaru virus** | **BEBV** | **59305** | **NC_016962** |
| **Chikungunya virus** | **CHIKV** | **37124** | **NC_004162** |
| **Getah virus** | **GETV** | **59300** | **NC_006558** |
| **Mayaro virus** | **MAYV** | **59301** | **NC_003417** |
| **O'nyong-nyong virus** | **ONNV** | **11027** | **NC_001512** |
| **Ross River virus** | **RRV** | **11029** | **NC_001544** |
| **Semliki forest virus** | **SFV** | **11033** | **NC_003215** |
| Barmah Forest virus | BFV | 11020 | NC_001786 |
| Eilat virus | EILV | 1231903 | NC_018615 |
| Middelburg virus | MIDV | 11023 | NC_024887 |
| Ndumu virus | NDUV | 59302 | NC_016959 |
| Salmon pancreas disease virus | SPDV | 84589 | NC_003930 |
| Sleeping disease virus | SDV | 78540 | NC_003433 |
| Southern elephant seal virus | SESV | 1159195 | NC_016960 |
| Tai Forest alphavirus | TFV | 1930825 | NC_032681 |

* ‘New World’ alphaviruses are marked with asterisk and ‘SFV Complex’ alphaviruses are written in bold.
