# Supplementary figures and images for: cRegions—a tool for detecting conserved cis-elements in multiple sequence alignment of diverged coding sequences
Source: PeerJ. 2019 Jan 10;6:e6176. doi: 10.7717/peerj.6176 (PMC6330207; doi:10.7717/peerj.6176)

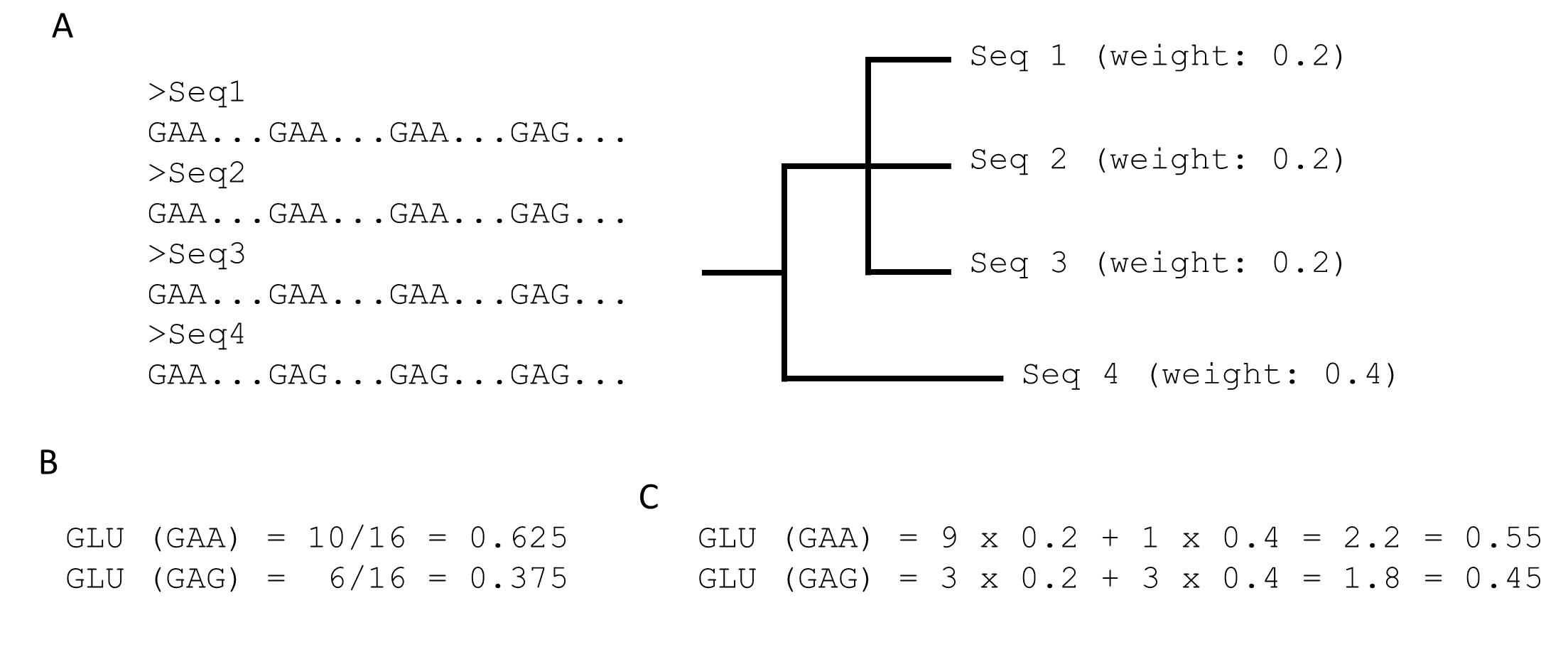

Supplement: Supplemental Information 2 — (A) Henikoff position-based sequence weights are calculated for each sequence based on the codon alignment. (B) Glutamic acid is encoded by two codons, therefore, the observed proportion for GAA is 0.625 and for GAG 0.375. (C) Henikoff position-based sequence weights are used to compensate for the over-representation of well-sequenced taxa in the multiple sequence alignment. The proportion for GAA and GAG are based on sequence weights. [file peerj-07-6176-s002.png]

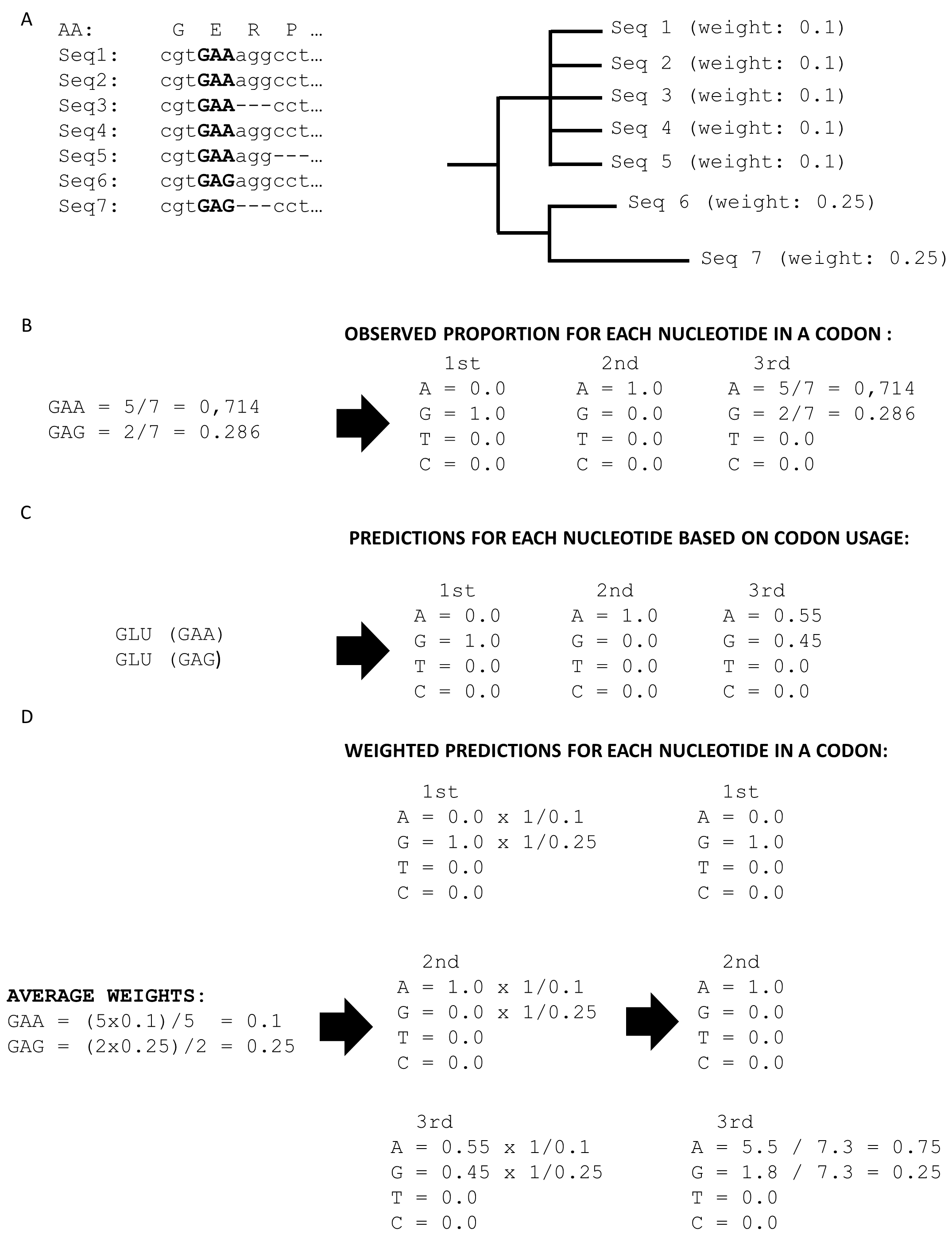

Supplement: Supplemental Information 3 — (A) Example dataset consists of 7 sequences. Five sequences are very similar; therefore, they have a low weight. Two sequences out of seven are different, therefore, having a high weight. (B) Glutamic acid is encoded by only two codons: GAA and GAG. Only the third position of a codon varies, therefore, including information. The observed proportion for the A nucleotide in the 3rd position of the glutamic acid codon is 0.714 and for G it is 0.286. (C) Predictions based on codon usage give us proportion for A is 0.55 and for G 0.45. By comparing observed and predicted proportions we will get a signal as there is a difference. However, it is a false positive signal due to a biased dataset. (D) Adjusting predictions with sequence weights, we can account for the over-representation of similar sequences. Only nucleotides that were observed are adjusted. [file peerj-07-6176-s003.png]

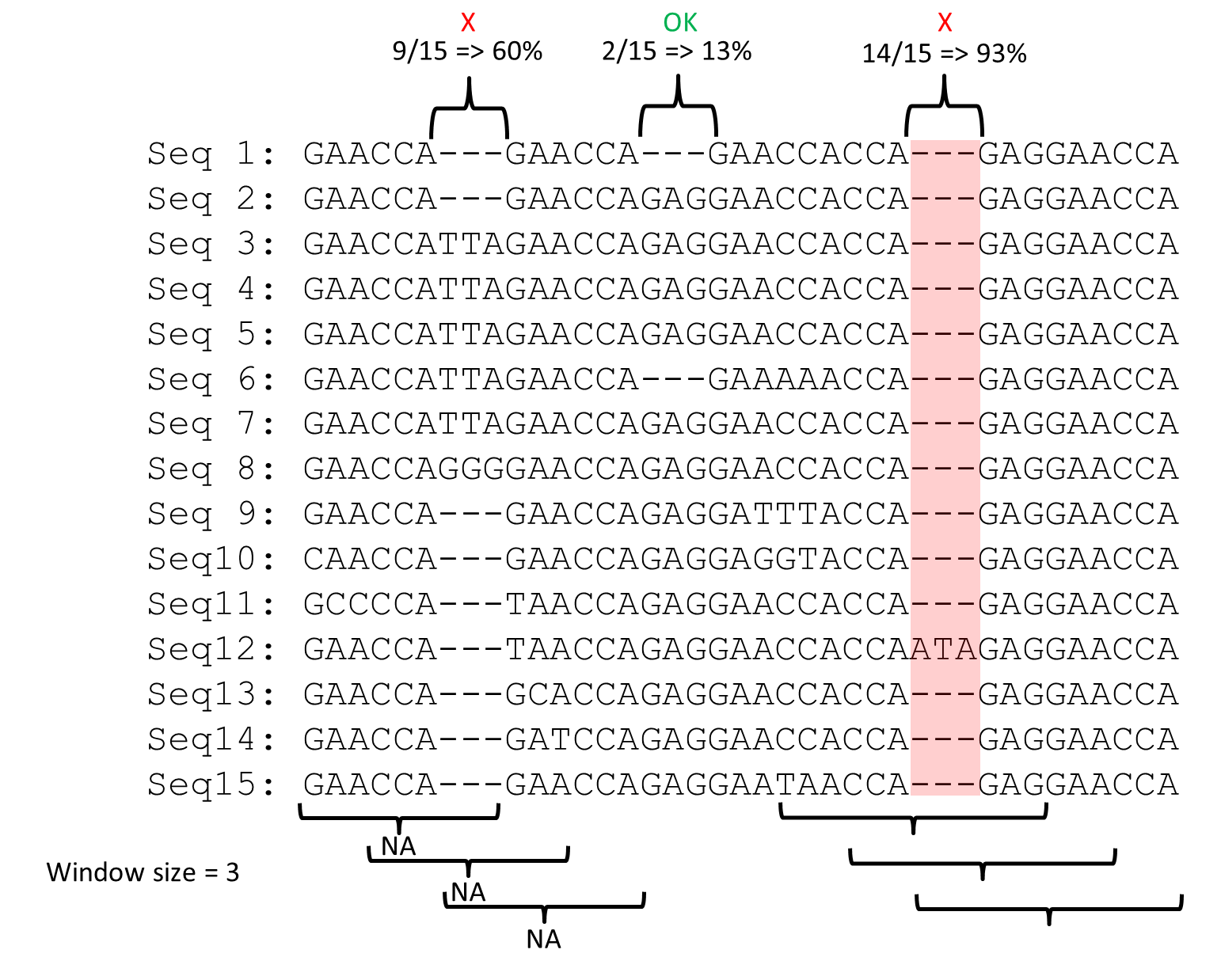

Supplement: Supplemental Information 4 — Allowed gaps parameter is a threshold (percentage of gaps in one column) if a metrics (RMSD, MAXDIF, CHISQ) should be calculated to a certain position. Metrics are not calculated at positions (red crosses) where the percentage of gaps in one column exceeds the threshold (percentage of allowed gaps). By default, a position (column) must have less than 20% of gaps. Skip gaps parameter is only used in sliding window mode. It is a threshold for skipping columns during sliding window mode instead of terminating the calculation as in the first three positions (NA). The threshold is used to avoid sliding window calculation termination while encountering insertions in a few sequences. By default, if the proportion of gaps in a position exceeds 90% (in other words, when insertion occurs in less than 10%) then this position is skipped (transparent red column) and next position is included to the window. [file peerj-07-6176-s004.png]

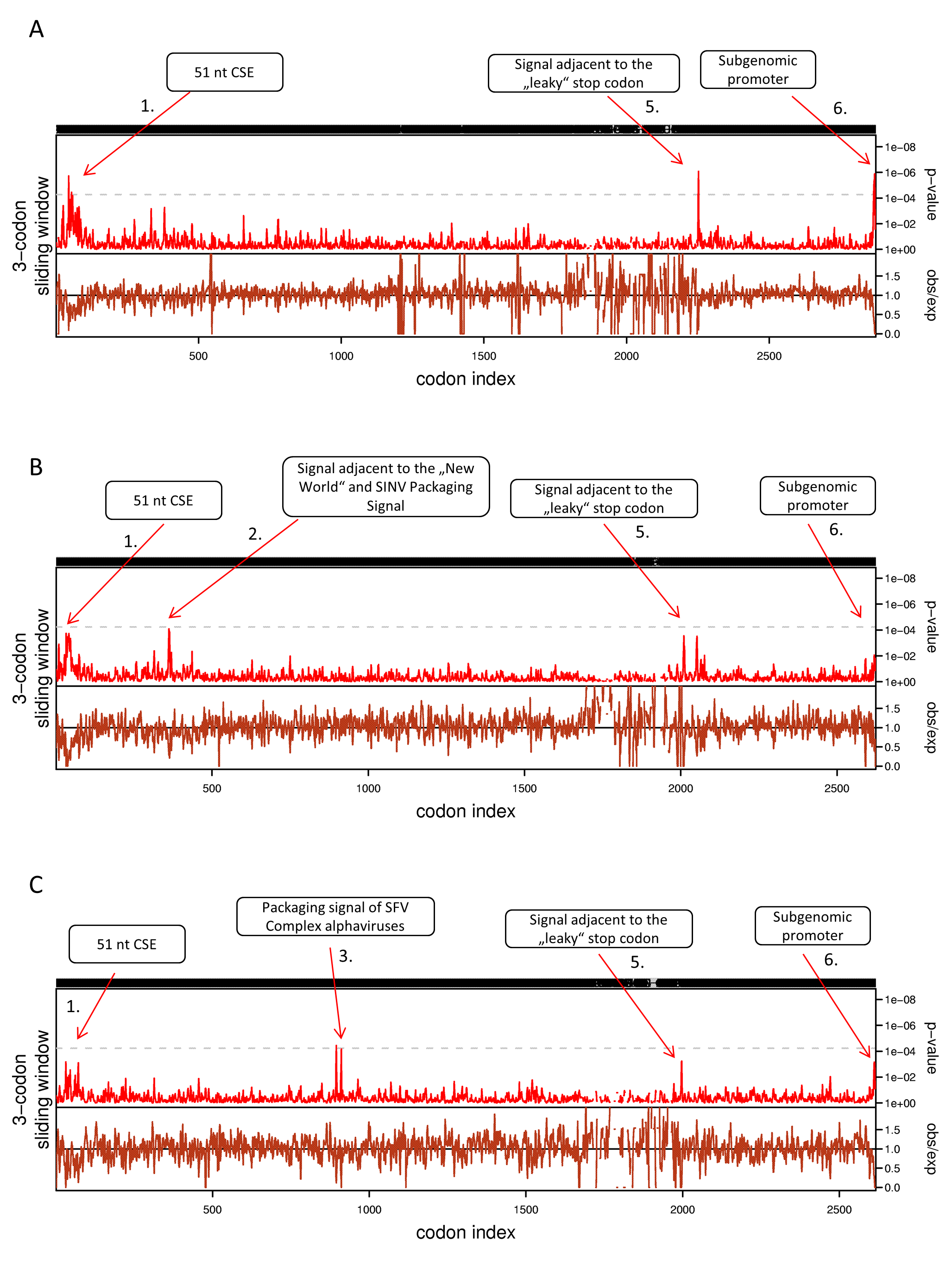

Supplement: Supplemental Information 5 — (A) Non-structural polyprotein alignment of all 24 Alphaviruses in our dataset. (B) Non-structural polyproteins of ‘New world’ Alphaviruses. (C) Non-structural polyproteins of ‘SFV Complex’ Alphaviruses. Non-structural polyprotein sequences were aligned with MAFFT using the default settings at http://www.ebi.ac.uk/Tools/msa/mafft/. Codon alignment was generated with pal2nal (http://www.bork.embl.de/pal2nal/). [file peerj-07-6176-s005.png]

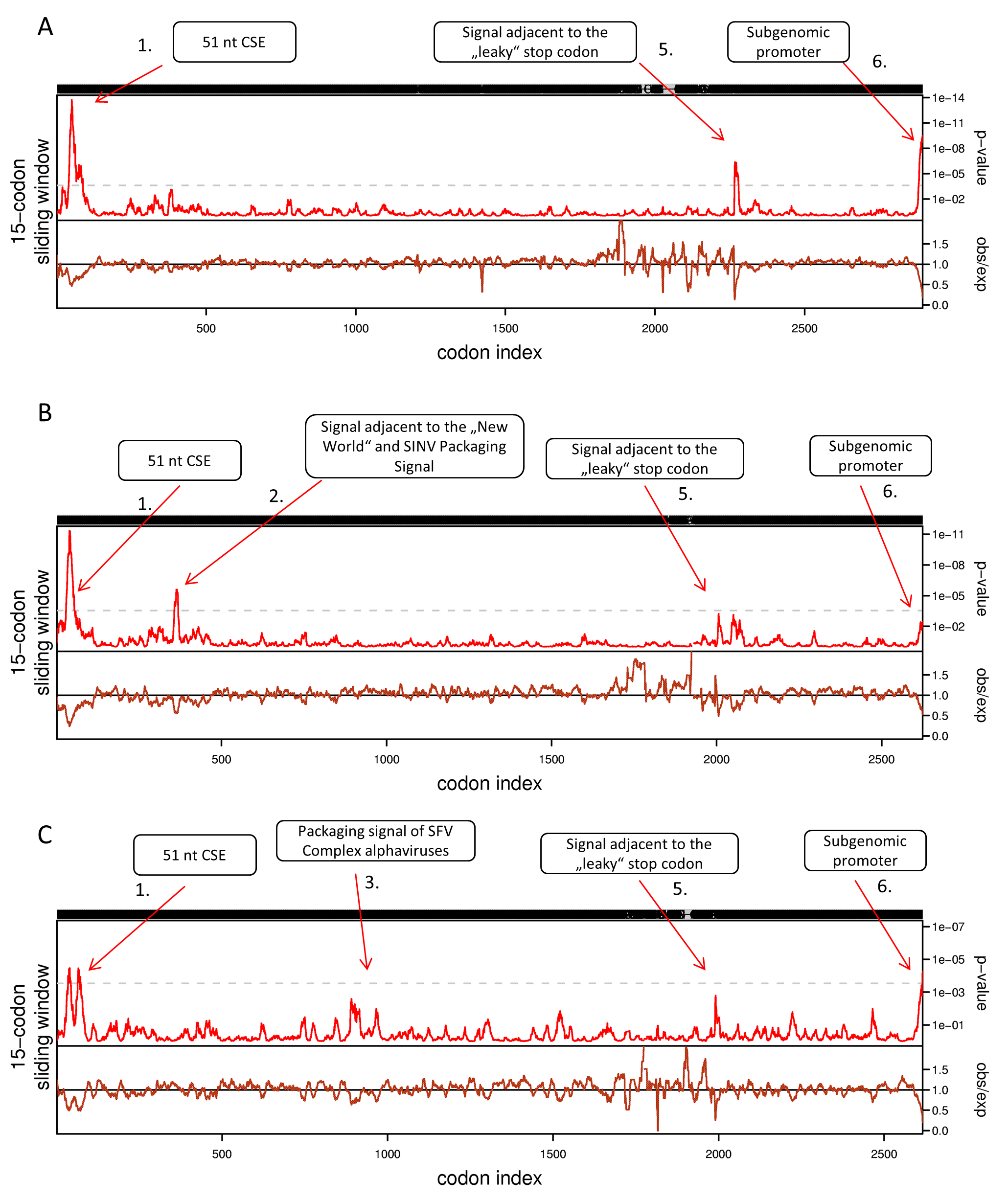

Supplement: Supplemental Information 6 — Same settings were used in the synplot2 publication (Firth, 2014). (A) Non-structural polyprotein alignment of all 24 Alphaviruses in our dataset. (B) Non-structural polyproteins of ‘New world’ Alphaviruses. (C) Non-structural polyproteins of ‘SFV Complex’ Alphaviruses. Non-structural polyprotein sequences were aligned with MAFFT using the default settings at http://www.ebi.ac.uk/Tools/msa/mafft/. Codon alignment was generated with pal2nal (http://www.bork.embl.de/pal2nal/). [file peerj-07-6176-s006.png]

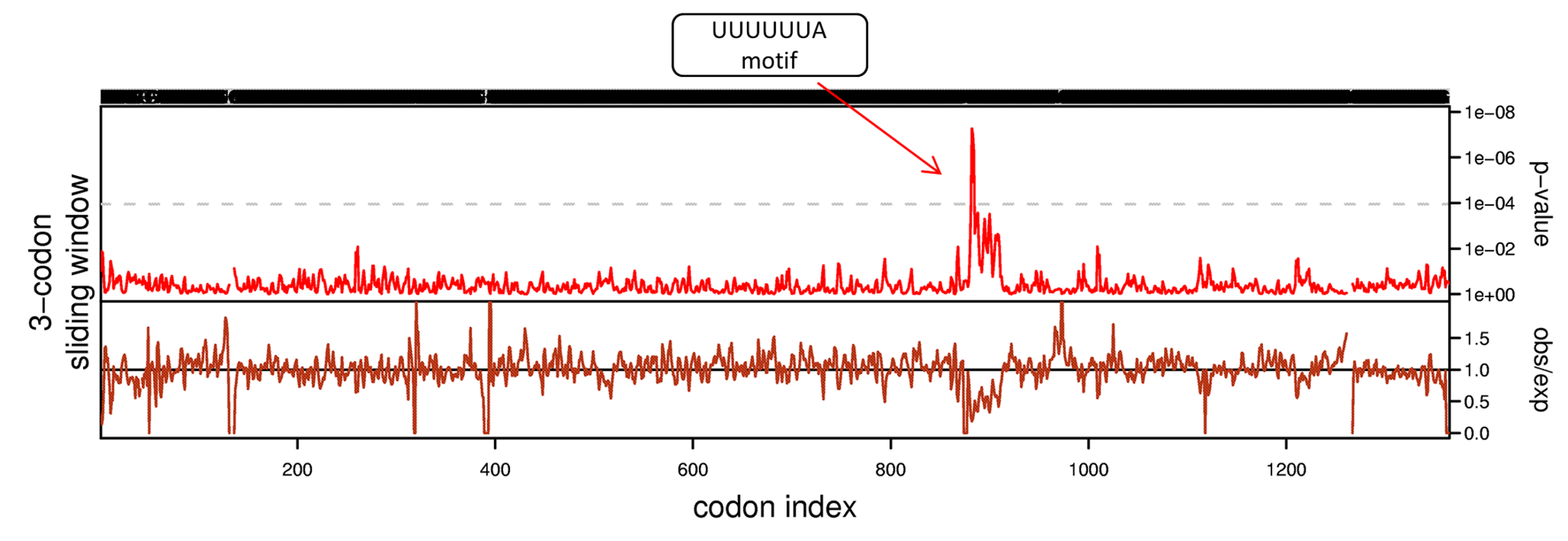

Supplement: Supplemental Information 7 — Significant signal was detected between codons 800–1000, which corresponds to a known UUUUUUA motif. The y-axis on the upper part of the synplot2 figure shows the p-value and the lower part shows obs/exp rato. Structural polyprotein sequences were aligned with MAFFT using the default settings at http://www.ebi.ac.uk/Tools/msa/mafft/. Codon alignment was generated with pal2nal (http://www.bork.embl.de/pal2nal/). [file peerj-07-6176-s007.png]

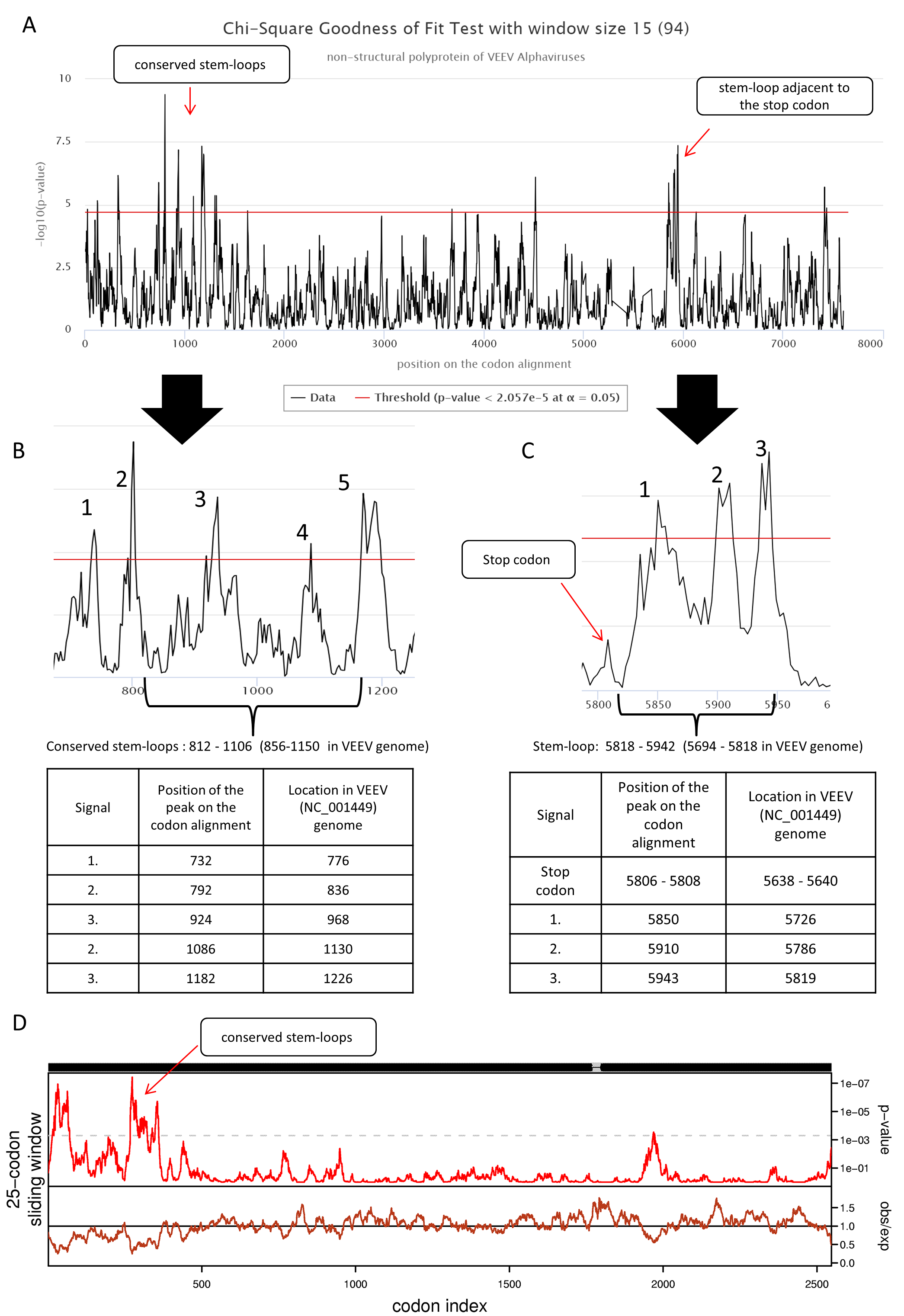

Supplement: Supplemental Information 8 — (A) Two known regions of RNA secondary structures were detected with cRegions using sliding window size 18. (B) Zoomed region of the conserved stem-loops. Locations of the signals in the VEEV genome are also provided (Kim et al., 2014). (C) Zoomed region of the stem-loops adjacent to stop codon. Locations of the signals in the VEEV genome are also provided (Firth et al., 2011). (D) Similar to the work done by Kim et al. sliding window size 25 was used with synplot2 (n = 12). Structural polyprotein sequences were aligned with MAFFT using the default settings at http://www.ebi.ac.uk/Tools/msa/mafft/. Codon alignment was generated with pal2nal (http://www.bork.embl.de/pal2nal/). [file peerj-07-6176-s008.png]

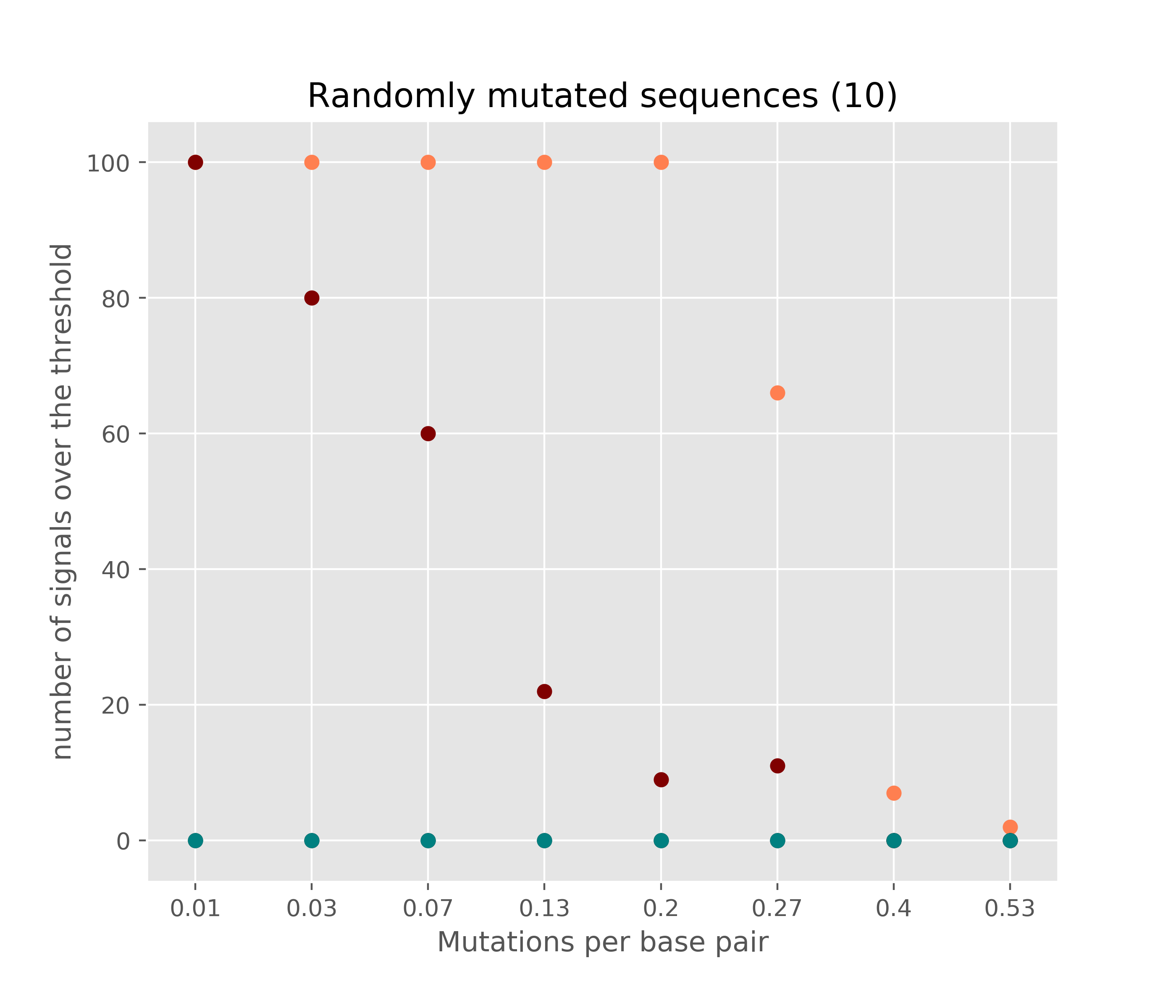

Supplement: Supplemental Information 9 — A random 3000nt long protein-coding sequence was created with SMS v2 tool (http://www.bioinformatics.org/sms2/random_coding_dna.html) (Stothard, 2000). A different number of random mutations (25–2000) were introduced into the original randomly generated protein-coding sequence 9 times with the SMS v2 mutate tool (http://www.bioinformatics.org/sms2/mutate_dna.html) (Stothard, 2000). Each simulated dataset consisted of 10 sequences each having the same number of mutations. Orange circles show the number of signals over the threshold without a threshold correction and using window size 1. Red circles show the number of signals with sliding window size 2. The deep blue-green (teal) shows the number of signals over the threshold when applying threshold correction. [file peerj-07-6176-s009.png]
